# Rapid Influenza Antigen Test for Diagnosis of Pandemic (H1N1) 2009

**DOI:** 10.3201/eid1605.091794

**Published:** 2010-05

**Authors:** Janice K. Louie, Hugo Guevara, Erica Boston, Melissa Dahlke, Maria Nevarez, Tong Kong, Robert Schechter, Carol A. Glaser, David P. Schnurr

**Affiliations:** California Department of Public Health, Richmond, California, USA

**Keywords:** Influenza, swine, pandemic (H1N1) 2009, viruses, respiratory infections, rapid antigen, expedited, dispatch

## Abstract

We compared the QuickVue Influenza test with PCR for diagnosing pandemic (H1N1) 2009 in 404 persons with influenza-like illness. Overall sensitivity, specificity, and positive and negative predictive values were 66%, 84%, 84%, and 64%, respectively. Rapid test results should be interpreted cautiously when pandemic (H1N1) 2009 virus is suspected.

Since its emergence, the pandemic (H1N1) 2009 virus has spread rapidly throughout the world. To diagnose influenza at the point of care, many clinicians rely on commercial rapid enzyme immunoassay tests, which are currently unable to differentiate between influenza A virus subtypes (*1*). Compared with PCR and viral culture, the sensitivity of rapid tests for seasonal influenza varies from 70% to 90% in children and <40% to 60% in adults (*2*,*3*). The positive and negative predictive values (PPVs and NPVs) of rapid tests depend on the prevalence of influenza viruses among the population being tested (*2*,*3*).

We compare PCR with a rapid influenza test to better characterize the diagnostic utility of the rapid test during the current pandemic. The QuickVue Influenza test (Quidel Corp., San Diego, CA, USA) detects influenza A and B viruses but does not distinguish between them. Clinicians may use the test in their offices because it is waived from Clinical Laboratory Improvements Amendment requirements based on documentation that test results by persons without formal laboratory training are in concordance with results by trained laboratorians.

## The Study

The California Department of Public Health (CDPH) supplied QuickVue Influenza test kits to clinicians participating in the Centers for Disease Control and Prevention (CDC) Sentinel Provider Influenza Surveillance Program. Sentinel providers performed the QuickVue Influenza test on a first respiratory specimen obtained from outpatients with influenza-like illness (fever >100°F and cough and/or sore throat) using the foam swab provided by QuickVue. Clinicians collected a second respiratory specimen using a sterile Dacron swab that was stored in viral transport media at 4°C for <72 hours before shipment to CDPH. Sentinel providers recorded information about patient demographics, symptoms, and QuickVue test results on a standardized specimen collection form.

At CDPH, specimens were tested by an influenza A universal real-time reverse transcription–PCR (rRT-PCR) assay with an analytical sensitivity (50% tissue culture infective dose /PCR input) of 0.51 for influenza A (*4*). If influenza A virus nucleic acid was detected, subtyping for human influenza A (H1 and H3) was performed. Specimens negative for any subtype were tested for pandemic (H1N1) 2009 by using a rRT-PCR detection panel provided by CDC. For all PCR testing, a cycle threshold (Ct, the cycle count at which amplified product yielded a detectable fluorescent signal) <40 was interpreted as positive. Sensitivity, specificity, predictive values, likelihood ratios, and posttest probabilities were estimated according to standard definitions (*5*). This activity was reviewed by the California Committee for the Protection of Human Subjects and determined to be a public health response that did not require institutional review board approval.

From May 4 to November 19, 2009, a total of 703 specimens were collected, including swabs from nares (293), nasopharynx (178), oropharynx (3), a mixture of sites (227), and unspecified sites (2). During this same period, statewide surveillance detected pandemic (H1N1) 2009 in 30%–50% of patients with influenza-like illnesses tested and 92%–100% of influenza viruses identified.

The median age of patients with influenza-like illness was 19 years (range 0–80 years). The median time from illness onset to specimen collection was 2 days (range 0–20 days). Of 703 specimens tested, 417 came from patients who had positive PCR results for influenza; 13 had seasonal influenza A subtypes, including 9 A/H1 and 4 A/H3; and 404 patients had pandemic (H1N1) 2009. Of these 404 patients, 266 (66%) had positive results and 138 (34%) had negative results by rapid antigen test (Table). Of 299 patients in which pandemic (H1N1) 2009 was not detected by PCR, 49 (16%) were positive and 250 (84%) were negative by the rapid antigen test. The prevalence of pandemic (H1N1) 2009 infection in all samples was 57%. The overall sensitivity, specificity, PPV, and NPV of the QuickVue Influenza Rapid Test for 2009 (H1N1) influenza when compared with PCR, regardless of the timing of collection, were 66%, 84%, 84%, and 64%, respectively, with a positive test result increasing the posttest probability from 57% to 84% and a negative test result decreasing it to 36%. The sensitivity, specificity, PPV, and NPV of the rapid test compared to PCR for persons <18 years of age were 68%, 80%, 87%, and 56%, respectively, and for persons >18 years were 64%, 86%, 82%, and 69%, respectively.

**Table:** Performance of rapid antigen test compared with PCR in the diagnosis of pandemic (H1N1) 2009*

Parameter	All specimens	Patient age <18 y†	Patient age >18 y†
No. rapid test positive, PCR positive	266	131	130
No. rapid test positive, PCR negative	49	19	28
No. rapid test negative, PCR positive	138	62	74
No. rapid test negative, PCR negative	250	78	166
Total no. tested	703	290	398
Prevalence of PCR positives in sample	0.57	0.67	0.51
Sensitivity	0.66	0.68	0.64
Specificity	0.84	0.80	0.86
Positive predictive value	0.84	0.87	0.82
Negative predictive value	0.64	0.56	0.69
Positive likelihood ratio (95% CI)	4.0 (3.1–5.2)	3.5 (2.3–5.3)	4.4 (3.1–6.3)
Posterior probability of positive test result (95% CI)	0.84 (0.81–0.88)	0.87 (0.82–0.91)	0.82 (0.76–0.87)
Negative likelihood ratio (95% CI)	0.41 (0.35–0.47)	0.40 (0.32–0.50)	0.42 (0.35–0.51)
Posterior probability of negative test result (95% CI)	0.36 (0.32–0.39)	0.44 (0.39–0.50)	0.31 (0.27–0.35)

*CI, confidence interval. †Does not include results for 15 case-patients where age was not recorded.

Ct values were available for 389 specimens in which pandemic (H1N1) 2009 virus was detected by PCR; of these, the median influenza A PCR Ct value was 26 for 135 specimens with a negative rapid test result and 21 for 254 specimens with a positive rapid test result (p<0.0001); samples with higher viral loads were more likely to be positive by rapid test (Figure). Even so, ≈25% of PCR-positive, rapid test–negative specimens had Ct values <23.

**Figure:**
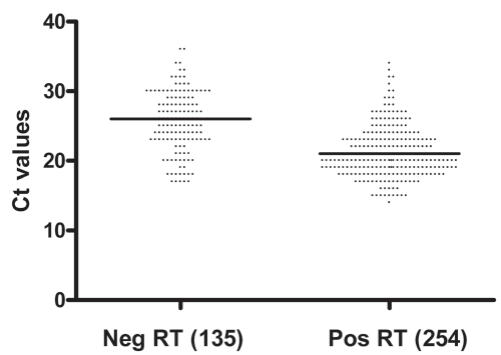
Comparison of cycle threshold (Ct) values for pandemic (H1N1) 2009 real-time reverse transcription–PCR-positive specimens (n = 389) with negative (neg) and positive (pos) rapid antigen test (RT) results. Solid lines represent median value for Ct.

Other smaller studies have found comparable sensitivities, but higher specificities, for rapid antigen tests for pandemic (H1N1) 2009. In a CDC study of 45 samples provided by state laboratories, the sensitivity of all rapid tests was 40%–69%, including 69% for QuickVue Influenza A+B (*6*). Others have found the QuickVue rapid tests to have sensitivities of 51%–63% and specificities of 99%–100% (*7*–*9*). During a large cluster of school outbreaks in New York, NY, USA, the sensitivity and specificity of the Binax NOW (Inverness Medical International, Bedford, UK) rapid test were 17.8% and 93.6%, respectively (*10*). As we found, positive rapid antigen test results in other studies also appear to correlate with higher concentrations of pandemic (H1N1) 2009 virus (*6*,*11*,*12*).

## Conclusions

Our findings illustrate the challenges clinicians face during the current pandemic. Because clinical symptoms of pandemic (H1N1) 2009 are nonspecific, definitive diagnosis requires confirmatory PCR testing, which, when available, often requires several days between specimen collection and reporting of results. Rapid antigen tests are the only current option for screening and diagnosis at the point of care. Current CDC guidelines recommend that high-risk and hospitalized infected patients be treated promptly with antiviral drugs and managed by using specific infection control precautions (*13*). Given the frequency of error found in this study, pandemic (H1N1) 2009 cannot be excluded solely because of a negative rapid antigen test result. Likewise, false-positive results, which would be expected to increase when the prevalence of influenza as a cause of influenza-like illness decreases, may result in unwarranted treatment and infection control measures that can be labor and resource intensive. Although rapid antigen tests are reported to have high specificity for seasonal influenza, our findings conflict with previous assumptions that rapid antigen tests are sufficiently specific to guide decisions about withholding antiviral treatment or chemoprophylaxis for pandemic (H1N1) 2009 (*2*).

A difference in swab types between rapid and PCR testing might have affected sensitivity of the rapid test results. Likewise, although influenza B virus was detected in only 9 (0.09%) of 10,367 specimens during the 7.5 months of statewide surveillance, some rapid test results may have been interpreted as falsely positive due to infection with influenza B.

In conclusion, we found the QuickVue influenza test had suboptimal sensitivity and specificity for the detection of pandemic (H1N1) 2009 during a period of increased prevalence in California. This finding suggests that rapid test results that may lead to changes in clinical management or public health intervention should be confirmed with PCR. A strength of our study is its reflection of typical testing practices in outpatient settings and the need for reconsideration of the clinical application of rapid test results. The development of more accurate point-of-care tests for seasonal and pandemic (H1N1) 2009 infection is urgently needed.
